# CerealsDB 2.0: an integrated resource for plant breeders and scientists

**DOI:** 10.1186/1471-2105-13-219

**Published:** 2012-09-03

**Authors:** Paul A Wilkinson, Mark O Winfield, Gary LA Barker, Alexandra M Allen, Amanda Burridge, Jane A Coghill, Keith J Edwards

**Affiliations:** 1 School of Biological Sciences, University of Bristol, Bristol, BS8 1UG, UK

**Keywords:** Wheat, Single nucleotide polymorphisms, SNPs, Database

## Abstract

**Background:**

Food security is an issue that has come under renewed scrutiny amidst concerns that substantial yield increases in cereal crops are required to feed the world’s booming population. Wheat is of fundamental importance in this regard being one of the three most important crops for both human consumption and livestock feed; however, increase in crop yields have not kept pace with the demands of a growing world population. In order to address this issue, plant breeders require new molecular tools to help them identify genes for important agronomic traits that can be introduced into elite varieties. Studies of the genome using next-generation sequencing enable the identification of molecular markers such as single nucleotide polymorphisms that may be used by breeders to identify and follow genes when breeding new varieties. The development and application of next-generation sequencing technologies has made the characterisation of SNP markers in wheat relatively cheap and straightforward. There is a growing need for the widespread dissemination of this information to plant breeders.

**Description:**

CerealsDB is an online resource containing a range of genomic datasets for wheat (*Triticum aestivum)* that will assist plant breeders and scientists to select the most appropriate markers for marker assisted selection. CerealsDB includes a database which currently contains in excess of 100,000 putative varietal SNPs, of which several thousand have been experimentally validated. In addition, CerealsDB contains databases for DArT markers and EST sequences, and links to a draft genome sequence for the wheat variety Chinese Spring.

**Conclusion:**

CerealsDB is an open access website that is rapidly becoming an invaluable resource within the wheat research and plant breeding communities.

## Background

The issue of food security (defined as the access to sufficient, safe, nutritious food to maintain a healthy and active life) has become a focus of concern for governments around the world and has moved steadily up the political agenda. The human population is expected to reach nine billion by 2050, and it has been estimated that cereal production will need to increase by 50% by this time if we are to meet the challenge of feeding ourselves and the animals upon which we are dependent [1]. The cereal crops wheat, maize and rice are the main source of nutrition for humans and domesticated animals [2]. Wheat, which accounts for approximately 30% of global cereal consumption [3], is of fundamental importance in this regard with an estimated 2010 harvest of 651 million metric tonnes [4]. Ensuring that yields of wheat increase to meet future needs has become an important focus in agricultural research. The remarkable increases in yield experienced during the period 1950 to 2000, which were based on traditional breeding techniques, have unfortunately been unable to keep up with the increased demand of a rapidly expanding world population. In addition, the relative increase in wheat yields has slowed towards the end of the twentieth century [5]. To help address this need plant breeders and scientists must embrace the modern molecular biological technologies that allow them a more targeted approach to plant improvement.

Single nucleotide polymorphisms (SNPs), which are single base pair changes in the DNA sequence, are the most common form of sequence variation between individuals of the same species. Because of their frequency in the genome, SNPs have become the marker of choice for marker assisted selection (MAS) of agronomic traits of importance such as disease and drought resistance [6]. In order to target genes at any position throughout the genome it is necessary to saturate the wheat genome with validated SNP markers that can be used in MAS. Through the use of next-generation sequencing (NGS), our laboratory is actively searching for SNPs in the wheat genome and developing PCR assays for these so that they can be used as molecular markers by plant breeders.

The development of NGS platforms and high-throughput genotyping methods has made it possible to rapidly characterise and validate thousands of putative SNP markers in wheat at relatively low cost [7,8]. A number of online databases already exist that link to SNP markers and other genomic resources, such as Gramene which is an online data resource for comparative genome analysis in the grasses [9] and GrainGenes, a database for genetic and genomic information about Triticeae species and their wild relatives [10]. Whilst these sites contain a wealth of information, they have been designed primarily for scientists. In order to maximise the utility and application of SNP markers for plant breeders, it is imperative that such information is made freely available and easily accessible to non-experts in molecular biology. This requirement for the dissemination of validated SNP marker data to plant breeders has provided the impetus for the development of the CerealsDB SNP database.

We have greatly extended the functionality of CerealsDB, which was originally constructed to store a dataset of 26,382 EST sequences [11], to include a number of searchable online databases relating to wheat genomics. Principally, the CerealsDB website is now aimed at those who wish to obtain information about SNP markers, for example, the sequence upon which they are based, the primers used for their PCR-based identification or SNP haplotype information of common UK varieties. The CerealsDB website also allows users to search the original CerealsDB EST sequences and the draft genome sequence of wheat variety Chinese Spring (assembled using Roche 454 technology) and to search for diversity array technology (DArT) markers that have been bin-mapped to chromosomes arms.

## Construction and content

### Implementation

CerealsDB was implemented using a MySQL relational database management system (RDMS) database (version 5.051b ) running on a MacOSX server and hosted using the Apache web server (version 2.2) with Perl and PHP scripts used for all data retrieval and output. MySQL was selected for the database, as it is one of the leading open source industrial strength RDMS, and matches the quality and performance of other major proprietary databases. MySQL also finds advantage in multi-layered server design with independent modules and fast execution process. The Apache HTTP server was adopted due to its reliability, relative ease of configuration and wide language interface support system.

**Figure 1:**
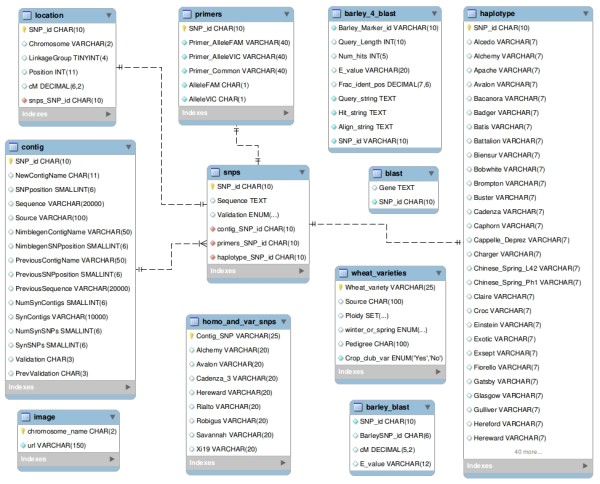
**Entity-relationship (ER) model for the cerealsDB SNP database, generated with MySQL workbench.** The schema displays the relationships between different tables within the SNP database.

CerealsDB is a relational collection of entities (tables) with appropriate attributes (fields) to define the wheat SNPs. An entity-relationship (ER) model for the SNP database is displayed in Figure 1. At present, the SNP database is composed of 11 tables of which four share an identifying relationship with the ‘Contig’ table through their common primary key, ‘SNP_id’. The ‘Contig’ table, which contains 111,442 records (representing putative SNP loci), consists of 16 attributes and includes information on those contigs that contain putative SNPs: e.g. source of the contig data, contig sequence and SNP position(s) within the sequence. The ‘Contig’ table also contains a field indicating whether the SNPs have been validated experimentally.

The ‘Location’ table consists of 3,813 entries and contains information with regard to chromosomal location and position in centimorgans (cM) of all the SNPs that have been mapped. The ‘Primers’ table, which has 6,337 records, contains the KASPar primer sequences (two forward and one reverse) for all the SNPs that have been experimentally assayed. The ‘Haplotype’ table includes 6,337 records and contains the SNP calls for all the validated SNPs against a panel of 101 wheat varieties. The ‘blast_url2’ table holds 37,837 entries that are linked to the contig table by the `NewContigName` attribute in a 1:1 identifying relationship. This table contains all hits reported when a BLASTX similarity search was performed for each contig versus the NCBI ‘embryophyta plant’ protein database [12]. Inclusion of this table allows users to search for SNPs on contigs that have a particular annotation (e.g., disease resistance). The ‘Source’ table cannot currently be queried by CerealsDB users and serves as an in-house resource. It contains 3,263 entries and holds information on the original source of a validated SNP and the location of the DNA on a sample plate stored in our laboratory.

The ‘Homo_and_var_SNPs’ (511,439 entries) and ‘VarietalSNPS’ tables (99,945 entries) are linked to one another by a 1:1 non-identifying relationship and are unlinked to any of the other tables. The ‘Homo_and_var_SNPs’ table contains information derived from a particular experiment designed to identify both homoeologous and varietal SNPs in eight wheat varieties (Alchemy, Avalon, Cadenza, Hereward, Rialto, Robigus, Savannah and Xi19): a homoeologous SNP is a sequence variation which exists between the different genomes (A, B and D) which make up the hexaploid wheat genome and a varietal SNP exists between wheat varieties and enables the differentiation of two varieties based upon that particular SNP location. The table, which contains 511,439 entries, contains a primary key which uniquely identifies the SNP and contig from which it has been derived, and the SNP calls, depth and value, for each locus for all eight varieties. This information has been published in Winfield et al. [13].

The ‘VarietalSNPS’ table contains information solely on those putative SNPs that have been classified as varietal; that is, SNPs that clearly distinguish one wheat variety from another. These varietal SNPs are of particular interest to breeders. This table is used principally for database searches used to populate the summary tables at the head of several web pages.

There are three stand-alone tables that are not linked to any other table in the database. The ‘WheatVariety’ table contains 101 records, one entry for each variety studied, and includes information on pedigree, winter/spring habit, and the original plant breeder for the 101 wheat varieties studied so far. The Images table (21 records) contains the URL to images of SNP maps of the 21 wheat chromosomes, and the table ‘IUPAC_codes’ holds 36 entries for translation of ambiguity codes.

The CerealsDB site has been extensively redesigned using cascading style-sheets (CSS) to standardise the format of the site, and improve the presentation, to provide a more intuitive browsing experience. Links are provided to the WheatBP educational resource and to other related sites that provide additional background information on wheat genomics.

### Data sources

SNP data was included from previous studies [14] as well as from ongoing experiments. SNPs were validated using the KBioscience Competitive Allele‒Specific PCR (KASPar) genotyping method which relies on the differential amplification of allele specific probes [15]. All SNP data were curated to remove redundancy and duplicate values. The contigs used in the database were generated from an assembly of wheat (5x Chinese Spring var.) cDNA sequences. Unique IDs were created for each contig using an 11-character code, a Bristol contig (BC) prefix for reference sequences followed by 9 digits (e.g., BC000000001). All unique SNPs were designated a 10-character code, beginning with a Bristol SNP (BS) prefix, followed by 8 digits (e.g., BS00000001). Cross-referencing and standardisation of file formats was performed using custom Perl scripts. The CerealsDB site also contains sequences from the draft assembly of the Chinese Spring genome, which is comprised of 5,321,847 contigs with a 5 x genome coverage. At this level of coverage, we would expect to have at least one read for > 95% of the genome.

The KASPar SNP database currently consists of data on 111,442 SNPs derived from 8 wheat varieties of which 99,945 are varietal SNPs (these are the SNPs that are of value to plant breeders) . Of these SNPs 4,986 have been validated (by screening against 101 varieties) and 3,813 mapped to a specific chromosomal location. Validated SNP entries contain information on location within a reference contig and primer sequences used for validation.

CerealsDB includes the original wheat EST database, containing 26,382 expressed sequence tags that were generated from hexaploid winter wheat (var. Mercia) cDNA and previously described by Wilson et al. [11]. These wheat ESTs can be searched by gene name or BLAST. There is also a database of DArT data that can be accessed through a clickable wheat ideogram; each chromosome image of the ideogram takes you to data related to that particular chromosome. DArT technology, which was developed as a hybridization-based tool for genotyping, incorporates many features of AFLP analysis combined with high throughput genetic analysis using a DNA microarray platform. We used deletion lines obtained from the Kansas Wheat Genetic and Genomic Resources Center (WGGRC) (http://www.k-state.edu/wgrc/Germplasm/Deletions/delindex.html) to assign DArT markers to chromosome arm specific bins. Of the 420 deletion lines held at the WGGRC, 74 lines were selected because they were the lines used in the NSF Wheat EST Genomics Project designed to assign wheat ESTs to chromosome bins. These lines possess homologous terminal deletions in single chromosome arms. These deletion lines (developed in the wheat variety Chinese Spring) provide powerful tools for the physical mapping of wheat chromosomes giving us the ability to map markers not only to specific homoeologous chromosomes but to assign them to bins of around 28 Mb [16] on specific chromosome arms. Whilst DArT markers are no longer used by plant breeders (having been superseded by SNP markers), this information is still relevant to the broader scientific research community.

## Utility and discussion

### Web interface and user querying capabilities

The CerealsDB website contains a range of datasets that are accessible via the menu bar and include the KASPar SNP database, a BLAST search page to query the Wheat genomic sequence, a download page for the draft wheat genome, a wheat EST database, a search page for wheat DArT data and a page describing the mapping of DArT markers using wheat deletion lines.

**Figure 2:**
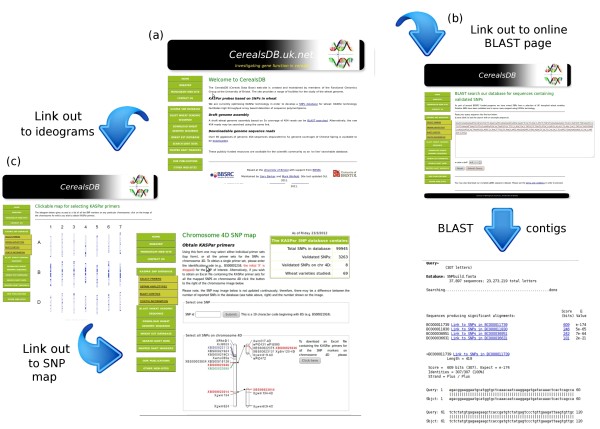
**The functionality of the KASPar SNP database is displayed with (a) the CerealsDB homepage, from which a range of pages are available for querying the SNP database.** For example, contigs containing SNPs can be searched by BLAST (**b**) and SNP maps can be dynamically retrieved from the database by clicking on ideograms (**c**) for a particular chromosome on one of the sub genomes.

The KASPar SNP database allows SNP name and contig queries. Some queries (e.g. SNP name) are exact. Sequence queries are BLAST similarity based [17]. Some of the functionality of the CerealsDB site is displayed in Figure 2. All SNPs listed in CerealsDB have been assigned a unique ID and the output page for a query gives information about the gene name and locus. All validated SNP information can be searched by chromosome and sub-genome and the resulting data is downloadable as an Excel spreadsheet.

The interface is designed to be simple and provide quick access for plant breeders who may not possess an in depth knowledge of genetics. Chromosome information is accessed via a clickable image (ideogram). These are arrayed on the screen according to the chromosome and sub-genome to which they belong. The initial page for the KASPar SNP database contains statistics on the SNPs within the database and also links to bar graphs showing the distribution across the 21 wheat chromosomes of all mapped SNPs for the mapping populations Avalon x Cadenza and Rialto x Savannah. In addition, there are also graphs showing the relationship between the expected number of mapped SNPs to each of the three genomes (A, B and D), based on their relative lengths, and the actual number mapped.

There are four main query roots for the KASPar SNP database:

1) “Select primers”; this can provide information on the primers for individual SNPs or for all the SNPs on a particular chromosome. Primers for SNPs on each chromosome can be selected by clicking on the relevant chromosome image of the ideogram, or by entering a specific SNP ID. Alternatively, all SNPs located on that particular chromosome can be downloaded as an Excel spreadsheet. Scrolling down the page reveals a map of the chromosome and the SNPs that are currently mapped to it.

2) “Obtain haplotypes”; select for information on the SNP alleles in the 101 wheat varieties against which the SNPs have as yet been validated. Clicking the relevant chromosome image of the ideogram will take the user to information on that particular chromosome. Alternatively, haplotypes can be selected for particular varieties on specific sub-genomes and chromosomes. Once again, results can be downloaded as an Excel spreadsheet.

3) “Blast contigs”; allows the user to input a nucleotide sequence and perform similarity searches (using BLAST) against the Bristol contigs. If the search identifies a contig and it contains SNPs, then the user can display the positions of each SNP within that particular contig.

4) “Contig information”; facilitates the search for information about the contig on which a SNP is found by providing a SNP ID, or the contig name to see whether it contains any SNPs. If a SNP is found, the output displays the contig sequence, SNP position, information on mapping and any associated BLAST annotation (the original BLASTX reports are also included). A field is provided to search for contigs based on a specific term, for example ‘disease resistance’ or ‘cold’, and will return all contigs that contain this term in the contigs’ associated BLASTX report.

The draft assembly of the gene-rich regions of the Chinese Spring genome, or the raw sequence reads can be searched using BLAST and a drop down field is available for setting the e-value cut-off.

Wheat ESTs can be searched by gene name, similarity search (via BLAST) or westdb cloneID. The wheat DArT markers can be searched by marker name or wheat line and the facility is available to download the DArT datasets in Excel format. Mapped DArT markers can also be viewed on their respective chromosome by clicking the appropriate chromosome image of the ideogram.

Finally, there is a Help/FAQ page for users, with answers to potential questions about the data in the database and other features on the website.

## Conclusions

Substantial enhancement has been made to the original CerealsDB site through the inclusion of databases for SNP and DArT markers as well as other genomic resources. In order to make CerealsDB comprehensible to non-scientists and to retain its relevance to scientific specialists, version 2.0 has improved both functionality and the user-friendliness of the web interface. As the SNP database grows in size and the number of KASPar validated SNPs increases, we expect traffic to the CerealsDB site to grow. The website has already attracted considerable interest from the plant-breeding and research communities and has recorded, on average, more than 5,600 unique visits per month over the past year (web traffic statistics generated by Webalizer version 2.23). Our laboratory is actively developing and validating SNP markers, and these are regularly added to the database. Future developments planned for the CerealsDB site include the linking of SNP data with phenotypic data and the integration of the KASPar SNP database with other cereal genomics databases. We aim to make CerealsDB an important, freely available resource by releasing all data into the public domain without restrictions. This will ensure that wheat breeders across the world have the data they require to create new varieties of wheat that will be needed to help feed the world beyond 2050.

## Availability and requirements

### Availability

CerealsDB can be accessed online from the following URL : http://www.cerealsdb.uk.net/CerealsDB/Documents/DOC_CerealsDB.php. The SNP database is publicly and freely accessible, requiring no registration and with no restrictions on use.

### Technical requirements

It is recommended that one of the following browsers is used: Mozilla Firefox 3 on Linux, Mac OSX or Windows, Internet Explorer 8 on Windows, Safari 4 on Mac OsX or Windows, Chrome on Linux or Windows.

## Abbreviations

BC, Bristol contig; BLAST, Basic local alignment search tool; BS, Bristol SNP; CSS, Cascading style sheets; DArT, Diversity array technology; ER, Entity-relationship; EST, Expressed sequence tag; FAO, Food and Agriculture Organisation of the United Nations; FAQ, Facts and questions; HTTP, Hypertext transfer protocol; ID, Identification; MAS, Marker-assisted selection; NCBI, National Center for Biotechnology Information; NGS, Next-generation sequencing; Perl, Practical extraction and report language; PHP, Hypertext pre-processor; RDMS, Relational database management system; SNP, Single nucleotide polymorphism; URL, Uniform resource locator.

## Competing interests

The authors declare that they have no competing interests.

## Authors' contributions

PAW and MOW wrote the manuscript. MOW, GLAB & PAW built CerealsDB version 2.0, AMA and AB carried out the SNP validation experiments, JC carried out NGS sequencing and KJE conceived of the study, participated in its design and coordination, and helped to draft the manuscript. All authors have read and approved the final manuscript.
